# CGRP and Migraine: What Have We Learned From Measuring CGRP in Migraine Patients So Far?

**DOI:** 10.3389/fneur.2022.930383

**Published:** 2022-07-27

**Authors:** Katharina Kamm

**Affiliations:** Department of Neurology, Ludwig-Maximilians-Universität München, Munich, Germany

**Keywords:** migraine, headache, calcitonin gene-related peptide, neuropeptide, trigeminal system

## Abstract

The multi-functional neuropeptide calcitonin gene-related peptide (CGRP) plays a major role in the pathophysiology of migraine. The detection of elevated CGRP levels during acute migraine headache was the first evidence of the importance of the peptide. Since then, elevated CGRP levels have been detected not only during spontaneous and experimentally induced migraine attacks but also interictally. However, the detection of CGRP in peripheral blood shows conflicting results. In this respect, alternative detection methods are needed and have been already proposed. This article summarizes what we have learned from studies investigating CGRP in jugular and peripheral blood and reviews the latest state of research concerning the detection of CGRP in saliva and tear fluid as well as their contribution to our understanding of migraine pathophysiology.

## Introduction

Migraine is a highly prevalent disorder with a complex pathophysiology involving the peripheral and central nervous system (1–4). Although many aspects of the pathophysiology remain elusive the importance of the trigemino-vascular system (TVS) with its peripheral and central afferents connecting intracranial vasculature and meninges to the brainstem plays a key role in the generation of migraine pain (5–7). Activation of the trigeminal system leads to release of vasoactive neuropeptides, in particular calcitonin gene-related peptide (CGRP), followed by neurogenic inflammation, nociceptive modulation and peripheral and central sensitization (4, 8). The importance of CGRP in migraine pathophysiology is highly supported by different research results:

(1) CGRP levels are elevated in ictal (during the migraine attack) and interictal (48–72 h headache and medication-free) migraine patients (2),
(2) CGRP levels are reduced after abortive and prophylactic treatment (2),
(3) CGRP can induce migraine-like headaches in migraine patients (9, 10),
(4) CGRP antagonists and CGRP antibodies are effective abortive and prophylactic migraine treatments, respectively (11, 12).

This article reviews our current understanding of CGRP in migraine pathophysiology. It focuses on studies investigating CGRP in different migraine states and discusses what we have learned from measuring CGRP as a marker for migraine.

## Calcitonin Gene-Related Peptide and the CGRP Receptor in the Nervous System

### Calcitonin Gene-Related Peptide

Calcitonin gene-related peptide (CGRP) is a 37 amino acid regulatory neuropeptide and potent microvascular vasodilator that was first described in 1982 (13). It belongs to the calcitonin family comprising calcitonin, adrenomedullin, adrenomedullin 2 (intermedin) and amylin (14). In humans, two forms, α-CGRP and β-CGRP, are described (15). They show structural similarity and share 94% homology as well as identical binding affinity and intensity (13, 16). Here, the term CGRP will be used, unless otherwise essential.

α-CGRP is located in the central and peripheral nervous system and is primarily produced and stored in Aδ- and C-fiber sensory neurons in the trigeminal ganglion (TG) and dorsal root ganglia (DRG) (16, 17). α-CGRP is produced *via* tissue-specific alternative splicing from the Calcitonin I gene on chromosome 11, that also gives rise to calcitonin (18). First, a pre-mRNA transcript of the Calcitonin I gene is produced, then exon 4 is spliced out and the transcript is translated in a 121 amino acid pro-hormone. Finally, it is cleaved in the mature 37 amino acid polypeptide and stored in dense-core vesicles for transport to axon terminals (19, 20). β-CGRP is found primarily in the enteric nervous system and pituitary gland. It stems from the Calcitonin II gene, also located on chromosome 11 (16, 21).

CGRP can be subdivided into four sections (16, 22, 23): the N-terminus end, consisting of seven amino acids, is a ring-like structure formed by a disulfide bond at amino acid 2 and 7 (16). It is responsible for receptor activation and affinity (23). Amino acids 8–18 form an α-helix, which is responsible for orientation of CGRP and efficient receptor binding (23, 24). Amino acids 19–27 are present as β- or γ-twist (22). Although little is known, this part seems to be involved in receptor binding. The C-terminus (amino acids 28–37) builds the binding epitope and interacts with the N-terminus of the CGRP receptor (16, 25).

### The CGRP Receptor

The Calcitonin family members bind to G-protein coupled receptors (GPCRs) to exert their actions (26).

The CGRP receptor is a membrane-bound heterodimer comprising the calcitonin receptor-like receptor (CLR) and the receptor activity modifying protein 1 (RAMP1) (16, 27, 28). Further, the two cytosolic proteins, receptor component protein (RCP) and the α-subunit of the GS protein (GαS) belong to the receptor complex. All components are needed to form a functional receptor which is distributed within the peripheral and central nervous system as well as the cardiovascular system (29).

The CLR is a member of the class B “secretin-like” family of G protein-coupled receptors (GPCR). It is structured in seven transmembrane-spanning domains with an extracellular N-terminus and a cytosolic C-terminus. By binding of CGRP to the N-terminus the signaling cascade is initiated (16).

RAMP1 belongs to the RAMP family including RAMP1, RAMP2, and RAMP3. It is specific to the CGRP receptor and consists of one transmembrane-spanning domain with a long extracellular N-terminal domain and a short intracellular C-terminus. It is responsible for high affinity binding of CGRP and receptor trafficking (30, 31).

If the CLR is combined with RAMP2 or RAMP3, respectively, receptors for adrenomedullin or adrenomedullin 2 are formed (32).

RCP is needed for signal transduction, in detail it connects the CLR and the cytosolic G protein-mediated signaling pathway leading to the production of cyclic adenosine monophosphate (cAMP) (2).

After CGRP binding, the receptor is phosphorylated and internalized (2, 26).

The AMY_1_ receptor formed by the calcitonin receptor (CTR) interacting with RAMP1 is another CGRP receptor (26, 32), however its physiological relevance needs to be determined (28). *In vitro*, both CGRP and amylin bind to the AMY1 receptor. Due to high potency of CGRP at this receptor and its widespread distribution a physiological role has been hypothesized (27).

## The Role of CGRP in Migraine Pathophysiology

The trigeminal nerve, the trigemino-vascular system (TVS) and the trigemino-cervical complex (TCC) play a pivotal role in the generation of migraine pain (5, 33). However, the origin of migraine attacks - whether peripheral or central - remains unclear (3, 34). Recent data suggest a central origin (35, 36), although this is beyond the scope of this review.

### The Trigeminal Nerve

Together with the ophthalmic, maxillary and mandibular branches (V1-3), the trigeminal nerve is the largest cranial nerve (V1: 26.000 fibers; V2: 50.000 fibers; V3: 78.000 fibers), responsible for tactile and pain perception of the face and the meninges as well as for motor control of masticatory muscles (37–39).

**Figure 1:**
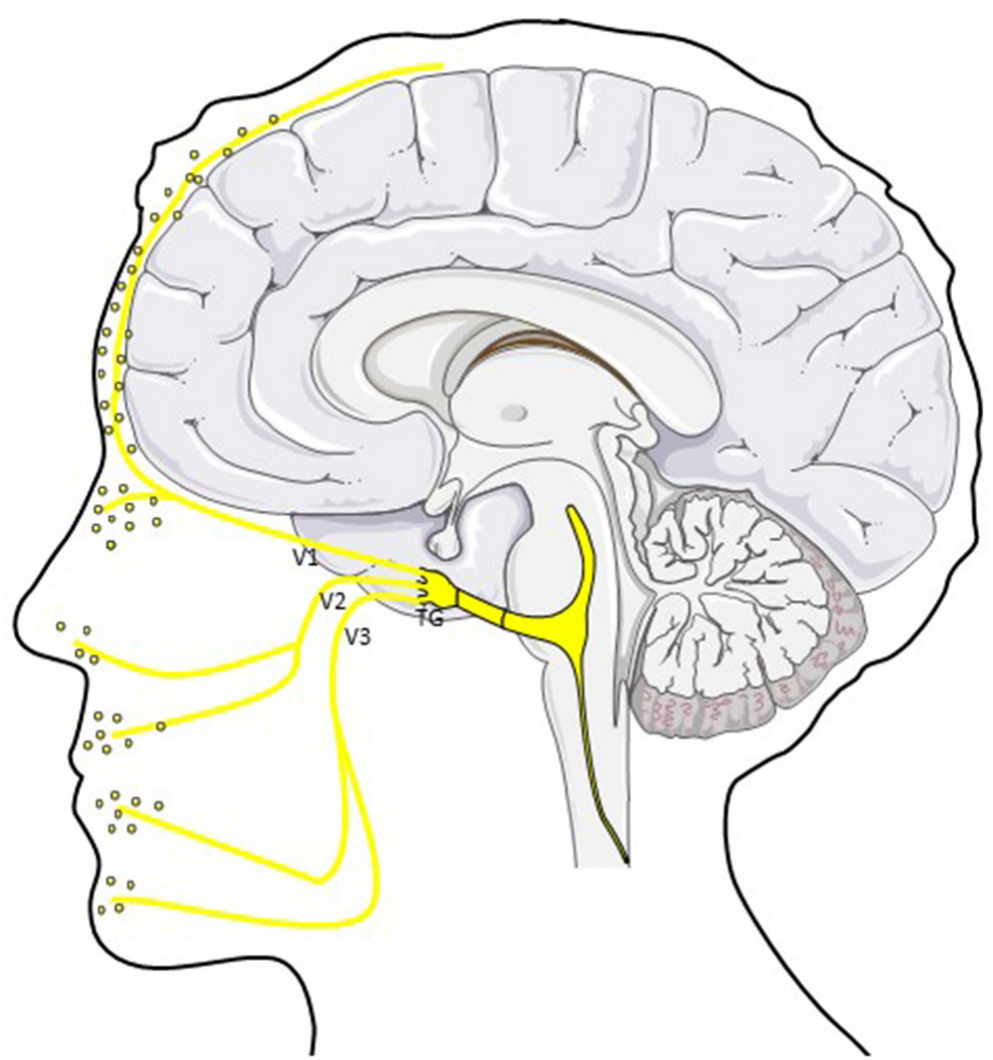
CGRP in the trigemino-vascular system. CGRP is released from peripheral afferents of the ophthalmic (V1), mandibular (V2) and maxillary (V3) division of the trigeminal nerve. Different studies showed elevated CGRP levels in saliva and tear fluid in ictal and interictal migraine patients (40). TG; trigeminal ganglion.

The ophthalmic division (V1) innervates the upper part of the face, most of the dura mater and cerebral vasculature (see Figure 1) (37, 39, 41). It terminates in the lacrimal, frontal and nasociliary nerve which again give rise to small sensory terminal afferents. Due to the major innervation of intracranial structures by the ophthalmic division, most nociceptive stimuli are conveyed by this branch to the trigeminal ganglion (TG) (39).

The maxillary division (V2) innervates sensitively the mid-part of the face including the upper lip and cheek. Sensitivity of the lower face and motor innervation of the chewing muscles is provided by the mandibular branch (V3) (37, 39).

The trigeminal ganglion (TG) consists of 20.000–150.000 pseudo-unipolar neurons with distal axonal branches forming the abovementioned divisions and a proximal axonal branch reaching the TCC in the brainstem (39, 42). Fifty percent of small- and medium-sized neurons show CGRP immunoreactivity (30, 43) primarily found in sensory neurons and their unmyelinated C-fibers or thinly myelinated Aδ-fibers, not in glial cells. CGRP is commonly colocalized with substance P (SP).

The TG also contains the CGRP receptor, however CGRP and CGRP receptor components are rarely co-expressed (30). CLR and RAMP1 are expressed in 40% of large neurons, satellite glial cells and in the wall of vessels of the TG (30, 44, 45).

TG neurons innervating intracranial vessels store several other neuropeptides like SP, neurokinin A/B, pituitary adenylate cyclase-activating peptide (PACAP), dynorphins, serotonin, amylin and glutamate which are also thought to be involved in migraine pathophysiology (37).

### The Trigemino-Vascular System

The trigemino-vascular system comprises the trigeminal nerve and its afferents to the intracranial vasculature and the meninges (46, 47). Nociceptive nerve fibers innervate pial, subarachnoid and dural blood vessels. Highest density of trigeminal fibers is found along proximal arteries and decreases in distal vessels, however, it was suggested that small cerebral vessels are also involved in pain (48–50). CGRP is also present in veins, although to a lesser degree. Due to low CGRP levels in blood, it was concluded that the peptide rather acts locally in the vessel wall (16).

Upon activation of the trigeminal system, CGRP and other neuropeptides like SP or PACAP are released from peripheral afferents and subsequently neurogenic inflammation occurs.

### Neurogenic Inflammation

Neurogenic inflammation is a neural-driven inflammatory process caused by the release of vasoactive neuropeptides. It is hypothesized to be a key mechanism of migraine pathophysiology (51–54) comprising plasma extravasation and vasodilation leading to nociceptor activation and sensitization (47, 55); Also, activated meningeal nociceptors lead to a release of vasoactive and proinflammatory peptides (55, 56). Nevertheless, the initiation of meningeal inflammation remains unclear. For example, activation by cortical spreading depolarization (CSD) or through the release of inflammatory mediators by mast cells is discussed (3, 4).

### The Trigemino-Cervical Complex

**Figure 2:**
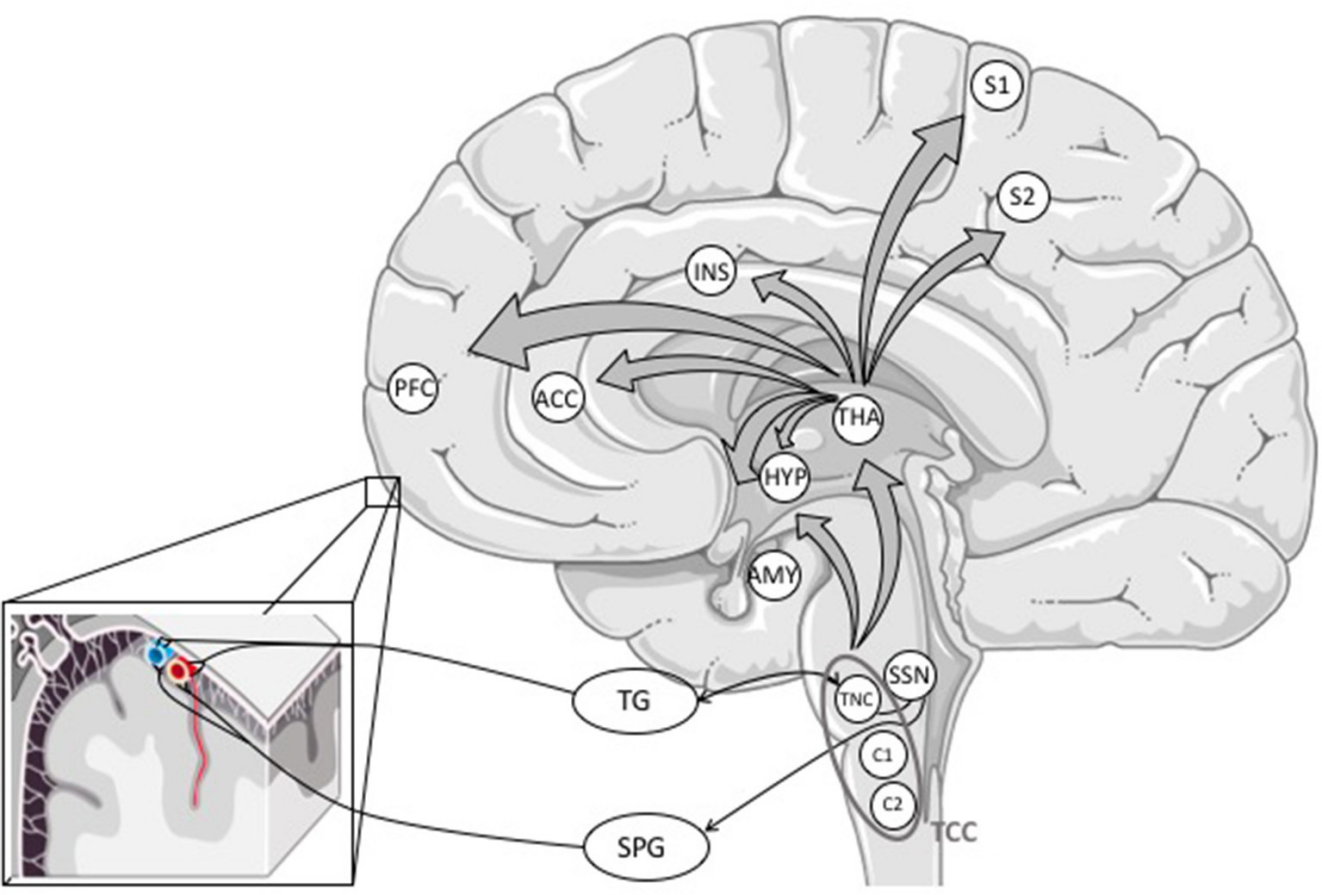
Primary afferents from the meninges and cerebral blood vessels reach the trigeminal ganglion, mostly through the ophthalmic branch (V1). The information is processed *via* first-order neurons in the TG to second-order neurons in the trigeminal nucleus caudalis forming the trigemino-cervical complex with C1 and C2 dorsal horns of the cervical spinal cord. The TCC projects to different areas in the brainstem (not outlined in this figure) and the thalamus. The activation of the TCC might also activate the trigeminal autonomic reflex. From the thalamus, nociceptive signals are conveyed to (sub-)cortical structures involved in pain perception (16). ACC, anterior cingulate cortex; AMY, amygdala; HYP, hypothalamus; INS, insula; PFC, prefrontal cortex; S1 and S2, somatosensory cortex; SPG, sphenopalatine ganglion; SSN, superior salivary nucleus; TCC, trigemino-cervical complex; TG, trigeminal ganglion; THA, thalamus; TNC, trigeminal nucleus caudalis.

Nociceptive signals from the meninges and intracranial vessels are transmitted mainly *via* the ophthalmic branch (V1) to first-order sensory neurons in the TG. From there, pain signals are conveyed to second-order neurons of the trigemino-cervical complex (TCC) in the brainstem consisting of neurons of the trigeminal nucleus caudalis (TNC) and C1 and C2 dorsal horns of the cervical spinal cord (see Figure 2) (5, 33).

The TCC projects to different areas in the brain stem and the thalamus where nociceptive signals are further processed (4, 5, 33, 57).

### Central Pain Pathways

From the thalamus, nociceptive signals are projected from third-order neurons to cortical and subcortical structures involved in pain perception (58), but thalamic nuclei are also involved in non-headache symptoms like photo- or phonophobia (33, 59).

In the CNS, pain signals are processed in the so-called pain matrix consisting of the primary and secondary somatosensory cortex, insula, anterior cingulate cortex and prefrontal cortex (4, 58).

## Investigations of CGRP in Migraine Patients

### Ictal CGRP—Investigations of CGRP During Spontaneous and Experimentally-Induced Migraine Attacks in Blood

In the late 1980s and early 1990s the neurovascular aspect of migraine pathophysiology was brought into focus (60, 61). Further, it became possible to investigate neuropeptides and their role in the innervation of cerebral vasculature and in migraine (62).

First, ictal migraine patients—referring to patients with a migraine headache at the time of study participation- and subsequently other migraine states like interictal or chronic migraine were investigated. These studies established and confirmed the importance of the trigeminal system and the neuropeptide CGRP in the pathophysiology of migraine. To date, only CGRP was reliably detected in migraine patients (6). An overview of studies investigating CGRP in blood, saliva and tear fluid gives Table 1.

**Table 1:** Overview of studies investigating CGRP in blood, saliva, and tear fluid.

	**Participants**	**Samples**	**Examination**	**Method**	**CGRP concentration**
**Plasma and serum**					
Goadsby et al. (62)	22 MWA/MOA patients (*f* = 16; 36 ± 13 y), HC	Plasma (EJV, CV)	Ictal	RIA Detection limit: 10 pmol/l	MWA EJV: 92 ± 11 pmol/l (vs. HC, *p* < 0.001) CV: 40 ± 6 pmol/l MOA EJV: 86 ± 4 pmol/l (vs. HC, *p* < 0.001) CV: 43 ± 6 pmol/l HC: < 40 pmol/l
Goadsby et al. (70)	8 migraine patients (*f* = 7; 34 ± 6 y)	Plasma (EJV)	Ictal, post-sumatriptan s.c. 3 mg	RIA Detection limit: 10 pmol/l	CGRP pre-treatment: 60 ± 8 pmol/l (*p* < 0.05) CGRP responder (*n* = 6): 40 ± 8 pmol/l
Gallai et al. (71)	45 MOA patients (*f* = 20; 16.3 ± 2.6 y), 30 MWA patients (*f* = 12; 15.4 ± 2.3 y), 20 HC (*f* = 15; 15.1 ± 2.1 y)	Plasma (CV)	Interictal (headache-free 48 h prior to blood sampling), ictal (2–4 h after migraine onset)	RIA Detection limit: 1 pmol/l	Interictal MOA: 34.7 ± 7.2 pmol/l (vs. HC, n.s.) MWA: 39.3 ± 8.6 pmol/l (vs. HC, n.s.) HC: 38.2 ± 6.5 pmol/l Ictal MOA: 51.4 ± 7.8 pmol/l (vs. interictal, *p* < 0.03) MWA: 50.3 ± 6.7 pmol/l (vs. interictal, *p* < 0.05)
Ashina et al. (78)	20 EM patients (*f* = 16; 40 ± 9 y), 20 HC (*f* = 12; 41 ± 14 y)	Plasma (CV)	Interictal (72 h medication- and headache-free prior to blood sampling)	RIA Detection limit: <1 pmol/l	EM: 75 ± 8 pmol/l (vs. HC, *p* = 0.005) HC: 49 ± 3 pmol/l
Juhasz et al. (73)	15 migraine patients (*f* = 15, 41.9 ± 2.3 y), 8 HC (*f* = 8, 38.5 ± 4.4 y)	Plasma (CV)	NTG-induced headache attack, blood sampling before and after headache	RIA Detection limit: n.a.	Migraine patients Basal, with headache: 20.2 ± 1.9 pmol/l (vs. without headache, *p* = 0.018) Basal, without headache: 14.0 ± 1.3 pmol/l Basal: 18.4 ± 1.7 pmol/l (vs. HC, *p* = 0.24), 1 h-post-headache onset: 22.2 ± 2.6 (vs. basal, *p* < 0.05) HC: 15.1 ± 2.0 pmol/l
Juhász et al. (72)	19 migraine patients (*f* = 19; 45 ± 1.4 y)	Plasma (CV)	NTG-induced headache attack, blood sampling before and after sumatriptan nasal spray	RIA Detection limit: n.a.	Sumatriptan responder (*n* = 6) Ictal: 16.9 ± 2.8 pmol/l (vs. 1 h-post suma, *p* = 0.034) 1 h post-sumatriptan: 14.7 ± 2.2 pmol/l Sumatriptan non-responder (*n* = 6) Ictal: 24.3 ± 2.5 pmol/l 1 h post-sumatriptan: 23.8 ± 2.4 pmol/l
Sarchielli et al. (76)	20 EM patients (n.a.)	Plasma (EJV)	Ictal, pre- and posttreatment with rizatriptan	RIA Detection limit: <1 pmol/l	Responder (*n* = 10) Pre-treatment: 12.2 ± 3.2 pmol/l Post-treament (2 h): 3.4 ± 1.1 pmol/l (vs. pre-treatment, *p* < 0.0001) Post-treatment (12 h): 2.1 ± 0.8 pmol/l (vs. pre-treatment, *p* < 0.0001) Non-responder (*n* = 10) Pre-treatment: 7.4 ± 2.4 pmol/l Post-treament (2 h): 7.9 ± 3.1 pmol/l (vs. pretreatment, n.s.) Post-treatment (12 h): 7.2 ± 3.1 pmol/l (vs. pretreatment, n.s.)
Tvedskov et al. (77)	21 EM patients (*f* = 17; 39 y)	Plasma (EJV, CV)	Interictal (headache- and medication-free 72 h), ictal	RIA Assay I: Detection limit: n.a. Assay II: Detection limit: <1 pmol/l	CGRP Assay I (EJV, *n* = 17) Ictal: 17.18 pmol/l (vs. interictal, *p* = 0.44) Interictal: 15.88 pmol/l CGRP Assay I (CV, *n* = 21) Ictal : 16.86 pmol/l (vs. interictal, *p* = 0.69) Interictal: 17.57 pmol/l CGRP Assay II (EJV, *n* = 17) Ictal: 32.59 pmol/l (vs. interictal, *p* = 0.42) Interictal: 30.59 pmol/l CGRP Assay II (CV, *n* = 21) Ictal: 33.37 pmol/l (vs. interictal, *p* = 0.43) Interictal: 31.84 pmol/l
Fusayasu et al. (79)	95 migraine patients (*f* = 77; 30.0 ± 10.4 y), 52 HC (*f* = 39; 29.2 ± 9.7 y)	Plasma (CV)	Interictal (headache-free 72 h)	EIA Detection limit: <4 pg/ml	Migraine patients: 19.0 ± 9.1 pg/ml (vs. HC, *p* < 0.01) HC: 13.4 ± 4.4 pg/ml
Rodríguez-Osorio et al. (80)	47 EM patients (*f* = 46; 37.8 ± 10.4 y), 23 HC (*f* = 22; 31.8 ± 11.0 y)	Serum (CV)	Interictal (Headache- and medication-free 72 h prior to blood sampling), ictal	ELISA Detection limit: n.a.	EM Interictal: 164.2 ± 139.1 pg/ml (vs. HC, *p* < 0.0001) Ictal (*n* = 19): 298.2 ± 100.3 pg/ml (vs. interictal *p* < 0.0001) HC: 37.1 ± 38.5 pg/ml
Cernuda-Morollón et al. (83)	103 CM patients (*f* = 103; 43.1 ± 11.7 y), 43 EM patients (*f* = 43; 44.4 ± 11.6 y), 31 HC (*f* = 31; 38.6 ± 12.8 y)	Serum (CV)	No medication 24 h prior and no headache at blood sampling	ELISA Detection limit: <4.3 pg/ml	CM: 74.90 ± 28.29 pg/ml (vs. HC, *p* < 0.001) EM: 46.37 ± 15.21 pg/ml (vs. HC, *p* < 0.005) HC: 33.74 ± 16.10 pg/ml
Cernuda-Morollón et al. (92)	81 CM patients (*f* = 77; 46.2 ± 11.0 y), 33 HC (*f* = 33; 39.4 ± 13.2 y)	Serum (CV)	Medication- 24 h prior and headache-free at blood sampling, treatment with OnabotulinumtoxinA	ELISA Detection limit: <4.3 pg/ml	CM 64.9 ± 31.0 pg/ml (vs. HC, *p* < 10^−10^) Responder (*n* = 61): 70.4 ± 31.9 pg/ml (vs. non-responder, *p* < 0.005) Non-responder (*n* = 20): 48.3 ± 21.2 pg/ml HC: 33.3 ± 15.7 pg/ml
Cernuda-Morollón et al. (85)	83 CM patients (*f* = 79; 44.2 ± 12.0 y)	Serum (CV)	Medication- 24 h prior and headache-free at blood sampling before and 1 month after OnabotulinumtoxinA treatment	ELISA Detection limit: <4.3 pg/ml	Responder (*n* = 64) Pre-treatment: 76.85 pg/ml (vs. non-res., *p* < 0.001) Post-treatment: 52.48 pg/ml (vs. pre-tr., *p* = 0.003) Non-responder (*n* = 19) Pre-treatment: 50.45 pg/ml Post-treatment: 51.89 pg/ml (vs. pre-treatment, n.s.)
Domínguez et al. (93)	62 CM patients (*f* = 60; n.a.), 24 HC (n.a.)	Serum (CV)	Medication- 48 h prior to and headache-free at blood sampling, treatment response to OnabotulinumtoxinA	ELISA Detection limit: n.a.	CM Responder (*n* = 47): 133.1 ± 86.6 ng/ml (vs. non-responder, *p* = 0.004) Non-responder (*n* = 15): 58.2 ± 91.7 ng/ml (vs. HC, *p* < 0.001) HC: 26.9 ± 12.5 ng/ml
Lee et al. (86)	99 EM patients [*f* = 78; 44 y (31–49)], 44 CM patients [*f* = 36; 39.5y (31–54)], 27 HC [*f* = 25; 34 y (27–42)]	Serum (CV)	EM: headache- and medication-free 24 h prior to blood sampling, CM: medication-free 24 h, headache-free at day of blood sampling	ELISA Detection range: 12.35–1,000 pg/ml	CM: 64.9 ± 15.32 pg/ml (vs. HC, *p* = 0.104) EM: 67.0 ± 20.70 pg/ml (vs. HC, *p* = 0.133) HC: 75.7 ± 20.07 pg/ml
Pérez-Pereda et al. (84)	101 CM patients (*f* = 89, 41 ± 10 y), 98 EM patients (*f* = 89, 41 ± 10 y), 97 HC (*f* = 88, 41 ± 10 y)	Serum (CV)	Interictal (medication- and headache-free 72 h prior to blood sampling)	ELISA Detection range: 12.35–1,000 pg/ml	CM: 18.02 pg/ml (14.4–24.7, vs. HC, *p* < 0.001) EM: 14.66 pg/ml (10.29–17.45, vs. HC, n.s.) HC: 13.99 pg/ml (10.10–17.87)
**Saliva**					
Nicolodi and Bianco (87)	15 migraine patients (*f* = 8; 43 ± 3.5 y), 34 HC (*f* = 18; 43.7 ± 4 y)	Saliva	Interictal (medication-free 72 h prior to blood sampling), ictal	RIA Detection limit: n.a.	Migraine patients Ictal: 27.3 ± 2.9 pmol/l (vs. interictal, *p* < 0.01) Interictal: 14.3 ± 2.5 pmol/l (vs. HC, *p* < 0.05) HC: 22.02 ± 1.7 pmol/l
Bellamy et al. (88)	5 migraine patients (n.a.), 5 HC (n.a.)	Stimulated saliva	Interictal (headache-free 72 h prior to blood sampling), ictal	RIA Detection limit: n.a.	Interictal: 53 pmol/mg total protein (vs. HC, *p* < 0.01) Ictal: 65 pmol/mg total protein 2 h-post-sumatriptan: 25 pmol/mg total protein (vs. ictal, *p* < 0.01)
Cady et al. (89)	22 EM patients (*f* = 20; 38.9 ± 2.7 y)	Stimulated saliva	Ictal, pre- and post-treatment with rizatriptan	RIA Detection limit: n.a.	Rizatriptan responder (*n* = 14) Basal: 51.1 ± 3.8 pmol/l total protein Rizatriptan non-responder (*n* = 8) Basal: 42.5 ± 4.0 pmol/l total protein
Jang et al. (82)	33 CM patients (*f* = 21; 43.7 ± 18.1 y), 36 HC (*f* = 19; 44.3 ± 14.2 y)	Saliva, plasma (CV)	n/a	EIA Detection limit: n.a.	CM Saliva: 431.6 ± 272.8 pg/ml (vs. HC, *p* = 0.026) Plasma: 253.6 ± 195.2 pg/ml (vs. HC, *p* = 0.003) HC Saliva: 301.5 ± 188.9 pg/ml Plasma: 136.2 ± 92.5 pg/ml
Cady et al. (90)	20 CM patients (*f* = 15; 48.5 ± 12.87 y)	Stimulated saliva	Interictal, pre- and 1 month post-Onabotulinumtoxin A	RIA Detection limit: n.a.	Pre-treatment: 39.4 ± 7.5 pg/mg total protein (vs. post-treatment, n.s.) Post-treatment: 25.5 ± 4.1 pg/mg total protein
Alpuente et al. (81)	22 EM patients (*f* = 22; 30.4 ± 9.4 y), 22 HC (*f* = 22; 31.2 ± 11.1 y)	Saliva, plasma (CV)	Interictal (headache-free 72 h prior to sampling), ictal	ELISA Detection limit: 0.39 pg/ml	EM Interictal: 98.0 (80.3) pg/ml (vs. HC, *p* = 0.034) Ictal: 247.0 (181.9–312.0) pg/ml HC: 54.3 (44.0) pg/ml
**Tear fluid**					
Kamm et al. (91)	48 EM patients (*f* = 42; 37.3 ± 12.0 y), 45 CM patients (*f* = 37; 34.4 ± 12.1 y), 48 HC (*f* = 33; 33.2 ± 9.6 y)	Tear fluid, plasma (CV)	Interictal (headache- and medication-free 72 h prior to sampling), ictal	ELISA Detection limit: 0.39 pg/ml	Migraine patients Interictal TF: 1.10 ± 1.27 ng/ml (vs. HC, *p* = 0.022) Interictal plasma: 6.32 ± 3.08 pg/ml (vs. HC, *p* = 0.528) Ictal, unmedicated TF: 1.92 ± 1.84 ng/ml (vs. interictal, *p* = 0.102) Ictal, medicated TF: 0.56 ± 0.47 ng/ml (vs. interictal, *p* = 0.011) HC TF: 0.75 ± 0.80 ng/ml Plasma: 6.57 ± 4.25 pg/ml

*CM, chronic migraine; CV, cubital vein; EIA, enzyme immunoassay; EJV, external jugular vein; ELISA, enzyme-linked immunosorbent assay; EM, episodic migraine; HC, healthy control; MOA, migraine without aura; MWA, migraine with aura; NTG, nitroglycerin; RIA, radioimmunoassay*.

In 22 patients with migraine with or without aura blood was collected during acute headache (with a median duration of 3 h) from the external jugular and cubital vein (62). CGRP levels were significantly elevated in migraine patients compared to healthy controls. Interestingly, elevated CGRP levels were only shown in blood drawn from the cranial circulation, but not in peripheral blood. Also, no changes in blood levels of SP, NPY, and VIP were detected.

The importance of the TG was further confirmed in 9 patients undergoing thermocoagulation—a therapeutic procedure to destroy tissue by heat produced by high-frequency electric currents—of the trigeminal ganglion for tic douloureux or atypical facial pain (63).

CGRP levels in the external jugular vein were elevated in patients with facial flushing, but otherwise not. The authors concluded that the activated trigeminal ganglion leads to elevated peptide levels (63).

If CGRP is intended to be used as a biomarker, it needs to serve as an objective disease measurement or indicator of a (patho-)physiological state (64–66). Several studies—especially studies measuring CGRP in the jugular vein- have shown increased CGRP levels during acute migraine and decreased CGRP levels after headache resolution. From these studies, it can be concluded that CGRP is a marker of the migraine attack. However, due to inconsistent findings in the overall studies, standardization of study procedures is needed to draw further conclusions (67–69).

Eight migraine patients treated a migraine attack with up to two doses of subcutaneous sumatriptan 3 mg (70). Blood was drawn from the external jugular vein during headache, before abortive treatment, immediately and 2 h after headache resolution. Six patients completely responded to the treatment and CGRP levels were significantly decreased after resolution of headache (70).

A further study investigated not only ictal, but also interictal CGRP levels in juvenile migraineurs compared to healthy controls (71). For interictal measurements migraine patients had to be headache-free for at least 48 h. During migraine attacks blood was drawn within 2–4 h after onset. Ictal CGRP levels were significantly elevated with maximum CGRP levels 2 h after onset. CGRP levels returned to baseline 2 h after spontaneous resolution, as shown in a subset of patients. Interestingly, no difference in CGRP levels was found in interictal migraine patients compared to controls (71).

CGRP levels were also investigated in experimentally-induced migraine attacks (72, 73). The application of nitroglycerin (NTG) is a common model to evoke a migraine-like headache in migraineurs (74, 75). Fifteen female migraineurs and eight healthy controls received nitroglycerin 0.5 mg sublingual. Cubital blood was drawn before NTG application and 60 and 120 min after beginning of a migraine-like headache. If no headache occurred, blood was drawn 5 and 6 h after drug administration (73).

As described before, an immediate headache occurred in a subset of migraineurs and controls and disappeared spontaneously, but no change in CGRP levels was seen during this headache. A migraine-like headache occurred in 2 of 8 controls and 10 of 15 migraine patients with a mean latency of ~6.5 h and a median intensity of 3.5 on the numerical rating scale (NRS). Migraineurs developing a headache showed significantly higher CGRP levels compared to patients without headache. Again, basal CGRP levels didn't show differences between the study groups.

In a following study, this research group investigated the influence of sumatriptan nasal spray 20 mg on CGRP levels in peripheral blood during an experimental migraine attack (72).

Nineteen female migraine patients developed a migraine-like headache attack after the application of sublingual nitroglycerin 0.5 mg. Cubital blood was drawn 120 min after migraine onset, immediately before and 60 min after sumatriptan application.

Based on the sumatriptan response, two groups were divided: patients (*n* = 6) who improved at least 30% showed significantly decreased CGRP levels, whereas patients (*n* = 13) who didn't improve accordingly showed no decrease in CGRP levels.

These study results were confirmed and extended by another study monitoring treatment response after rizatriptan in 20 EM patients (76). During six consecutive migraine attacks, the efficacy of rizatriptan was clinically screened. Ten responder (significant pain reduction within 2 h after rizatriptan intake and no headache recurrence within the next 48 h) and 10 non-responder (no significant reduction in pain intensity within 24 h after rizatriptan intake) were chosen.

During a spontaneous migraine attack, patients reached the headache center within 2 h and the external jugular vein was immediately catheterized. CGRP levels were analyzed at the time of catheterization and 1, 2, 4, 6, and 12 h after triptan administration. Ictal CGRP levels were significantly higher in treatment responder compared to non-responder. Further, CGRP levels were significantly reduced in responder, already after 1 h, but even more after 2 h and stayed at this level during the 12 h observation period. CGRP levels in non-responder didn't change significantly over the course of the migraine attack (76).

In spite of these study results, one study didn't find any CGRP level differences in external jugular or antecubital blood ictally and interictally (77). Patients enrolled in this study called the study team at the beginning of a migraine attack, restrained from taking acute medication and blood was drawn within 60 min after initial contact.

Interictal CGRP levels were investigated when patients were headache and abortive medication-free for 72 h. The study group used 2 CGRP assays, however no ictal and interictal differences in CGRP levels could have been detected.

### Interictal CGRP—Investigation of CGRP During Headache-Free Periods

The aforementioned study results highlighted CGRP as a potential marker of trigeminal activation as the neuropeptide is elevated during migraine attacks and reduced after headache resolution (2). From these study results it can be concluded that the neuropeptide represents different (patho-)physiological states of a migraine attack and can be used as biomarker. Further studies investigated interictal CGRP levels and the possible role of CGRP as a biomarker for migraine itself. Moreover, other and less invasive methods were examined and patient groups were chosen due to headache frequency and abortive medication intake.

#### Interictal CGRP in Episodic Migraine Patients

In interictal episodic migraine (EM) patients elevated (78–80) as well as unchanged CGRP levels were found in peripheral blood compared to healthy controls (71, 73, 77, 81).

The majority of the studies included migraine patients being headache- and abortive medication-free 72 h prior to blood drawing. No correlation between migraine attack frequency and CGRP levels was found (78).

#### Interictal CGRP in Chronic Migraine Patients

Different studies detected elevated CGRP levels in peripheral blood of chronic migraine (CM) patients compared to healthy controls (82), but also compared to EM patients (83, 84). The studies used different headache- and medication free periods which makes a comparison of the study results difficult (see Table 1).

However, another study investigating serum CGRP levels didn't find differences in CM patients and healthy controls. Migraine patients were headache-free 24 h prior to investigation (86).

#### Interictal CGRP as Treatment Response Marker

CGRP was analyzed as a potential marker for treatment response in CM patients (85, 92, 93). Eighty-three and, respectively, eighty-one CM patients received at least two injections of OnabotulinumtoxinA (155–195 units) following the PREEMPT protocol (94); treatment responder were defined as patients with a ≥50% reduction of headache episodes and a ≥50% subjective benefit (85) or as moderate (reduction of headache episodes and subjective benefit between 33 and 66%), respectively, excellent responder (reduction of headache episodes and subjective benefit > 66%) (92). CGRP levels were determined before and 1 month after OnabotulinumtoxinA administration.

77%, respectively, 75% of CM patients were considered responder. In both studies, pretreatment CGRP levels were significantly higher in responder compared to non-responder. CGRP levels decreased significantly after 1 month in the responder group, whereas this reduction could not have been detected in non-responder (85).

### Other Sources of CGRP for the Investigation in Migraine Patients

Due to the innervation of the trigeminal nerve and its ophthalmic, maxillary and mandibular branches other sources for detecting CGRP have been investigated in migraine patients (38, 39).

In general, saliva and tear fluid receive increasing attention as diagnostic fluids and to date, few studies have investigated CGRP in saliva and tear fluid (see Table 1) (95).

Former studies detected CGRP in human tears and changes in these peptide levels are hypothesized to represent (patho-)physiological alterations (96). The eye -more precisely the cornea, conjunctiva, meibomian and lacrimal glands- is innervated by sensory, sympathetic and parasympathetic nerves originating in the TG, the superior cervical and pterygopalatine ganglion, respectively (97–99). The cornea is highly innervated by CGRP-positive fibers from the ophthalmic branch (V1) (98), whereas the CGRP-positive innervation of the meibomian and lacrimal glands seems to be scarce (97, 99). Saliva is mainly produced by the parotid, submandibular and sublingual glands as well as numerous minor glands located in the submucosa of the mouth (100). Salivary glands are controlled by the autonomic nervous system, and to a lesser degree innervated by CGRP-positive fibers that evoke salivary secretion (101–103).

Advantages of the measurement of CGRP in these compartments might be higher neuropeptide concentrations due to direct innervation and a non-invasive and easy sample collection which enables repetitive measurements.

### CGRP in Saliva

#### Salivary CGRP Levels in Episodic Migraine Patients

Salivary CGRP levels were first investigated in 15 migraineurs compared to 34 healthy subjects in 1990 (87). Saliva was obtained ictally and interictally when patients had restrained from taking abortive medication for 72 h.

Significantly elevated CGRP levels were detected ictally, whereas lower CGRP levels were detected in interictal migraine patients compared to healthy controls (87).

Stimulated salivary CGRP levels were shown interictally and ictally in five EM patients compared to five healthy controls (88). Patients had to be headache-free 72 h prior to interictal investigation. After rinsing the mouth, saliva production was stimulated using 2% citric acid applied to the tongue. Initial saliva was discarded in order to avoid mixing unstimulated and stimulated saliva and 5 mL saliva was sampled. Saliva collection has been trained in the clinic and was performed independently by patients at home.

As shown before, the intake of sumatriptan 100 mg reduced ictal CGRP levels compared to unmedicated patients. In contrast to the above mentioned study results, interictal CGRP levels were significantly elevated in migraine patients compared to healthy controls. Importantly, no changes in peptide levels were detected between sampling in the clinic and at home (88).

These study results were extended by monitoring CGRP levels over the course of a spontaneous migraine attack in 22 EM patients by the same study group (89).

Compared to baseline CGRP levels, no change in CGRP levels during premonitory phase could have been detected, but during the occurrence of a mild or moderate headache. After intake of rizatriptan and headache resolution salivary CGRP levels were found to be near baseline levels. As shown before, triptan responder showed a significant increase of ictal CGRP levels, whereas non-responder didn't show significant changes in salivary CGRP levels during the migraine attack.

The authors further differentiated two groups: one group (*n* = 6) showed already elevated CGRP levels during the premonitory phase, sustained during the headache phase. In contrast, the other group (*n* = 8) showed highest CGRP levels during a moderate headache.

In a recent study salivary CGRP levels were continuously monitored and investigated interictally and ictally (81). Twenty-two EM patients and twenty-two healthy controls were included. For interictal sampling patients had to be headache-free for 72 h, in every participant peripheral blood was drawn interictally once. Saliva was independently sampled by patients and stored at home. Interictal saliva levels were significantly elevated in EM patients compared to healthy controls, whereas no significant difference was detected in CGRP plasma levels. Forty-nine migraine attacks were monitored by taking saliva samples at headache onset, after 2 and 8 h.

Again, ictal CGRP levels were elevated. Dependent on CGRP levels, the authors stated CGRP-independent and CGRP-dependent migraine attacks with significantly higher CGRP levels. Eighty percent of migraine attacks were CGRP-dependent and 20% were CGRP-independent. Relating to patients, 13 of 22 migraine patients showed only CGRP-dependent, 3 of 22 patients showed exclusively CGRP-independent attacks and 6 patients showed both types of migraine attacks. These study results support the above mentioned results as they indicate that several neuropeptides might be involved to different degrees in a migraine attack.

#### Salivary CGRP Levels in CM Patients

Salivary CGRP levels in chronic migraine is less investigated. One study showed significantly elevated CGRP levels in 33 CM patients compared to 36 healthy controls in resting whole saliva and periperhal blood (82).

#### Salivary CGRP as a Treatment Response Marker

To date, one study investigated salivary CGRP levels in 20 CM patients receiving OnabotulinumtoxinA compared to placebo (90). At inclusion, baseline salivary CGRP levels were determined and patients were divided in two study groups: group A received OnabotulinumtoxinA as described in the PREEMPT protocol (94), group B received saline. After 4 months treatment regimens were switched. Patients were instructed to obtain monthly saliva samples. In both study groups, headache days were significantly reduced after treatment, whereas the reduction of headache days was greater after OnabotulinumtoxinA treatment.

In the OnabotulinumtoxinA group, CGRP levels decreased at month 2 and 3, although this change didn't reach significance which the authors ascribe to little patient number. A respective decrease of CGRP levels was not detected after saline.

### Tear Fluid CGRP in EM and CM Patients

In our study group, tear fluid CGRP levels were investigated in 48 EM, 45 CM and 48 healthy controls (91). Interictal (no headache and abortive medication in the last 48 h) and ictal migraineurs visiting our outpatient headache center were continuously included. Tear fluid was sampled using a plastic capillary located at the lateral canthus of both eyes. Blood was drawn from the cubital vein.

In general, we found CGRP levels to be about 140× higher in tear fluid compared to plasma levels. Further, tear fluid CGRP levels were significantly elevated in interictal migraine patients compared to healthy controls. No differences in tear fluid CGRP levels could have been detected in episodic and chronic migraine patients. One explanation for this finding might be the high frequency of migraine days in EM patients.

As shown before, ictal migraine patients who had restrained from taking abortive medication 48 h prior to investigation showed highest CGRP levels, although this only showed to be a trend which is likely due to little patient number (*n* = 13).

Ictal migraine patients with intake of acute medication 48 h prior to investigation showed significantly reduced CGRP levels in tear fluid compared to interictal and unmedicated ictal patients.

## Discussion

Measuring CGRP in migraine patients has led to a better understanding of pain in migraine pathophysiology as well as it laid the foundation of the development of new abortive and prophylactic treatments (40). Further, the neuropeptide is recognized as a marker for the acute migraine attack and it might be a marker for migraine itself which could help to objectify the diagnosis in the future (67).

However, results of CGRP measurement in peripheral blood remain conflicting as well as comparability and reproducibility is often limited (69, 104).

Most probably, the differences in study results are caused by using distinct methods and inhomogeneous study groups (69).

Recent studies used more controlled inclusion and exclusion criteria, e.g., concerning ictal or interictal migraine or monthly headache frequency. Differences between interictal and ictal migraine patients have been shown in several studies and reduced CGRP levels were detected after intake of abortive medication up to 12 h in blood and 48 h in tear fluid (76, 91).

To date, little is known concerning the influence of monthly migraine frequency. There are studies showing increased CGRP levels in CM compared to EM patients (83, 84), however other studies didn't find significant differences (86, 91) or a correlation with number of the headache days (78).

In this respect, the analysis of CGRP levels in chronic migraine might be especially challenging since headache- and medication-free periods are scarce due to ≥15 headache days/month (105).

However, the investigation of rigorous subgroups concerning headache days, associated symptoms or distinct clinical factors, but also comorbidities, age and gender will contribute to our understanding of CGRP. Further, interferences of the above mentioned factors with the peptide could be investigated which might also explain missing comparability of study results (67, 68).

Almost all studies used different study methods concerning blood drawing, processing and analysis as well as many study protocols haven't been sufficiently described. Thus, direct comparison of the studies is not possible and might be one of the most important explanations of different research results as well as limited reproducibility (69). CGRP is rapidly degraded with a short half-life of 7–9 min (106). This rapid degradation was proposed to cause negative study results in studies with longer processing times (104). Preparation of pre-chilled vials, the application of peptidase inhibitors [although the effect of peptidase inhibitors was also questioned (104)] and storage on ice until immediate processing needs to be carefully considered (69, 104). In this respect, the analysis of CGRP in plasma might be beneficial compared to serum.

Also, various analysis methods like radioimmunoassay (RIA), enzyme immunoassay (EIA) or enzyme-linked immunosorbent assay (ELISA) as well as the implementation of these procedures will affect reproducibility (68). This question was recently addressed and suggestions were proposed for standardization of study protocols (69).

After release, the neuropeptide is thought to be taken up by post-capillary veins and can subsequently be detected in the circulation (69). However, CGRP levels are low in blood and dilution needs to be considered (16). This is especially important if blood is taken peripherally with a wide distance from the location of release.

Alternative approaches like CGRP measurement in saliva or tear fluid has been proposed and their potential role in determination of the neuropeptide has been shown (81, 87, 88, 91). Advantages of these methods are higher CGRP concentrations due to direct innervation which might allow to detect even subtle differences in CGRP levels. CGRP-dependent and CGRP-independent migraine attacks as well as different CGRP levels over the course of a migraine attack have been detected in saliva. In the future, this might lead to a better understanding of the contributing neuropeptides or different expression patterns of these in different patient subgroups.

In this respect, the identification of molecule profiles might be an interesting approach for the future (67).

Further, the proposed sampling techniques are easy applicable and even self-administered sampling is possible. As it has already been shown this gives the opportunity to conduct longitudinal studies in real-life conditions and larger patient number might be easier to recruit since these sampling methods are well accepted by participants.

## Conclusion

Taken together, the detection of CGRP in migraine patients has enormously enhanced our understanding of migraine pathophysiology and provided new treatments. To enhance this knowledge, higher standardization of study protocols is needed in order to provide better comparability and reproducibility.

## Author Contributions

The author confirms being the sole contributor of this work and has approved it for publication.

## Conflict of Interest

The author declares that the research was conducted in the absence of any commercial or financial relationships that could be construed as a potential conflict of interest.

## Publisher's Note

All claims expressed in this article are solely those of the authors and do not necessarily represent those of their affiliated organizations, or those of the publisher, the editors and the reviewers. Any product that may be evaluated in this article, or claim that may be made by its manufacturer, is not guaranteed or endorsed by the publisher.
